# The Deceptive Shadow: Inverted Left Atrial Appendage Mimicking Thrombus After Infant Open Heart Surgery

**DOI:** 10.7759/cureus.89768

**Published:** 2025-08-11

**Authors:** Sachin Talwar, Shrividya Rao, Navnita Kisku, Lamak Kadiyani, Akhsya K Bisoi, Vishal V Bhende

**Affiliations:** 1 Cardiothoracic and Vascular Surgery, All India Institute of Medical Sciences, New Delhi, IND; 2 Cardiology, All India Institute of Medical Sciences, New Delhi, IND; 3 Pediatric Cardiac Surgery, Bhanubhai and Madhuben Patel Cardiac Centre, Shree Krishna Hospital, Bhaikaka University, Karamsad, IND

**Keywords:** congenital heart disease, intra-cardiac clot, intra-cardiac mass, left atrial appendage, thrombus

## Abstract

Inversion of the left atrial appendage (LAA) is a rare postoperative complication following cardiac surgery, often misdiagnosed as thrombus or vegetation on imaging, potentially leading to inappropriate management. We report a case of a five-month-old female infant who underwent elective surgical closure of a perimembranous ventricular septal defect via a right atrial approach under cardiopulmonary bypass. On postoperative day 2, transthoracic echocardiography (apical four-chamber view) revealed a 1 × 0.9 cm echogenic mass attached to the anterior mitral leaflet in the left atrium. The mass caused progressive mitral inflow obstruction, and by postoperative day 5, the patient developed acute pulmonary edema. Although initially suspected to be a thrombus, the diagnosis of LAA inversion was confirmed intraoperatively. Given the elongated nature and prolapse of the appendage into the mitral orifice, external ligation and clipping were chosen to ensure complete exclusion and prevent recurrence. Postoperative echocardiography demonstrated complete resolution of the mass and unobstructed mitral inflow, with sustained clinical recovery. This case highlights the importance of considering LAA inversion in the differential diagnosis of left atrial masses following congenital heart surgery. Early recognition and appropriate surgical management can prevent life-threatening complications.

## Introduction

Inversion of the left atrial appendage (LAA) is a rare postoperative complication, with fewer than 15 cases reported in the English literature, predominantly as isolated case reports [1-3]. It is most frequently observed following open-heart surgery and is often misinterpreted on echocardiography as thrombus, vegetation, or even tumor, potentially leading to unnecessary anticoagulation or surgical intervention. This diagnostic pitfall has been reported with both transthoracic echocardiography (TTE) and transesophageal echocardiography (TEE), especially when imaging planes are suboptimal or when inversion mimics a mass near the mitral valve [2-4].

The pathophysiological mechanism is thought to involve negative intra-atrial pressure during cardiac surgery, particularly during cardiopulmonary bypass (CPB) weaning. Excessive suction from left atrial venting, overzealous manual de-airing, or abrupt changes in intracardiac volume may create a vacuum effect that pulls the thin-walled LAA into the left atrial cavity. Pediatric patients, especially infants, are believed to be at increased risk due to reduced atrial wall stiffness and relatively larger LAA size compared to the atrial chamber. The condition has been described following atrial septal defect (ASD) and ventricular septal defect (VSD) repairs, as well as during the use of mechanical circulatory support such as left ventricular assist devices (LVAD) [5-7].

We describe this occurrence in an infant who had experienced a perplexing postoperative course following surgical repair of a ventricular septal defect (VSD). The LAA inversion manifested as a left atrial mass on postoperative echocardiography and resulted in progressive mitral valve inflow obstruction, culminating in acute pulmonary edema. This case is noteworthy due to its delayed symptomatic presentation, initial misdiagnosis as thrombus, and successful management using external ligation and clipping of the LAA without the need for left atrial re-entry. It underscores the critical importance of considering LAA inversion in the differential diagnosis of postoperative intracardiac masses and highlights the role of intraoperative imaging and tailored surgical strategies in managing this rare but potentially life-threatening complication.

## Case presentation

A female infant was diagnosed with VSD at five months of age. She was admitted for elective VSD closure at the age of 5 months, weighing 7 kg. She had no known syndromic associations or comorbidities. Preoperative echocardiography revealed a perimembranous, nonrestrictive VSD with mild mitral regurgitation. No intracardiac masses, vegetations, or thrombi were identified.

Intraoperatively, standard hypothermic cardiopulmonary bypass (CPB) at 28 °C was initiated using aorto-bicaval cannulation with the placement of a right superior pulmonary vent. Cardioplegia was administered via a single dose of Del Nido solution at the aortic root following cross-clamping. The VSD was closed through a right atrial (RA) approach using a 4 mm × 6 mm Dacron patch (Bard Sauvage Filamentous Knitted Polyester fabric, AZ, USA). The procedure was uneventful. Intraoperative TEE confirmed successful VSD closure with no residual shunt or intracardiac mass. No abnormality was noted in the left atrial appendage during immediate post-CPB TEE.

On postoperative day 2, while still in the intensive care unit (ICU), she underwent TTE, which demonstrated moderate left ventricular dysfunction, mild mitral regurgitation, and an unexpected finding: a 1 × 0.9-cm echogenic mass attached to the anterior mitral leaflet in the left atrium (LA). Although cross-sectional imaging (MRI or CT) could not be performed due to logistical reasons, suspecting a thrombus, standard heparin infusion (10 units/kg/min) was initiated empirically to prevent embolic complications, which was later transitioned to oral warfarin. Hematologic investigations for potential coagulopathy revealed no abnormalities, and blood cultures were negative for bacterial growth. Despite serial evaluations, there was no change in the size of the echogenic mass. Contrast-enhanced tomography was deferred due to impaired renal function in the immediate postoperative period. By postoperative day 5, the infant’s clinical condition deteriorated with the development of acute pulmonary edema. Echocardiography revealed mitral valve inflow obstruction caused by the mass. Careful review of serial TTE images raised suspicion of an inverted LAA due to its shape and dynamic movement with the cardiac cycle.

**Figure 1:**
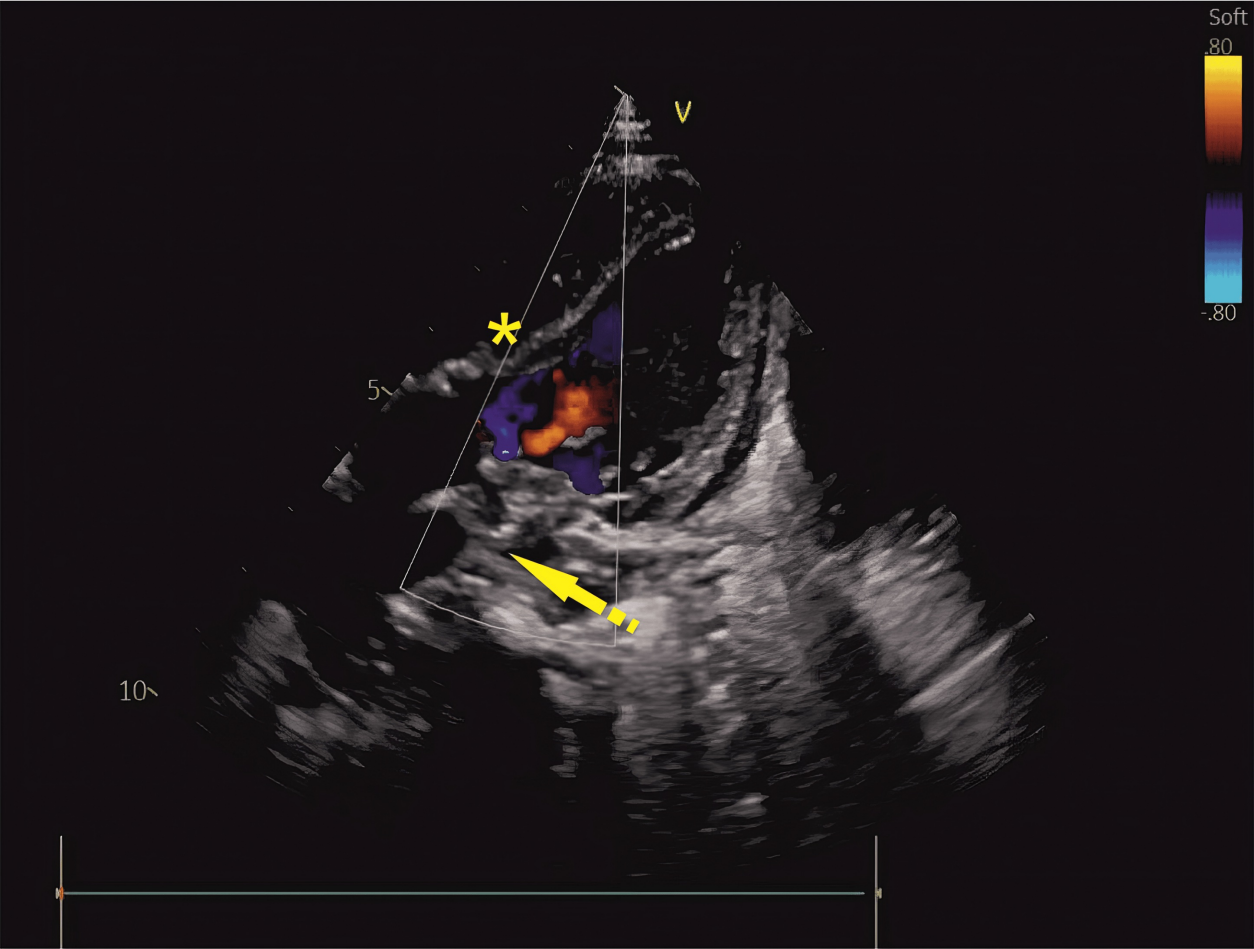
Two-dimensional echocardiography, apical four-chamber view The arrow indicates the left atrium, with a discrete echogenic mass abutting the mitral valve leaflet. The asterisk (*) denotes the left atrial cavity. Image Credits: Dr. Lamak Kadiyani

**Figure 2:**
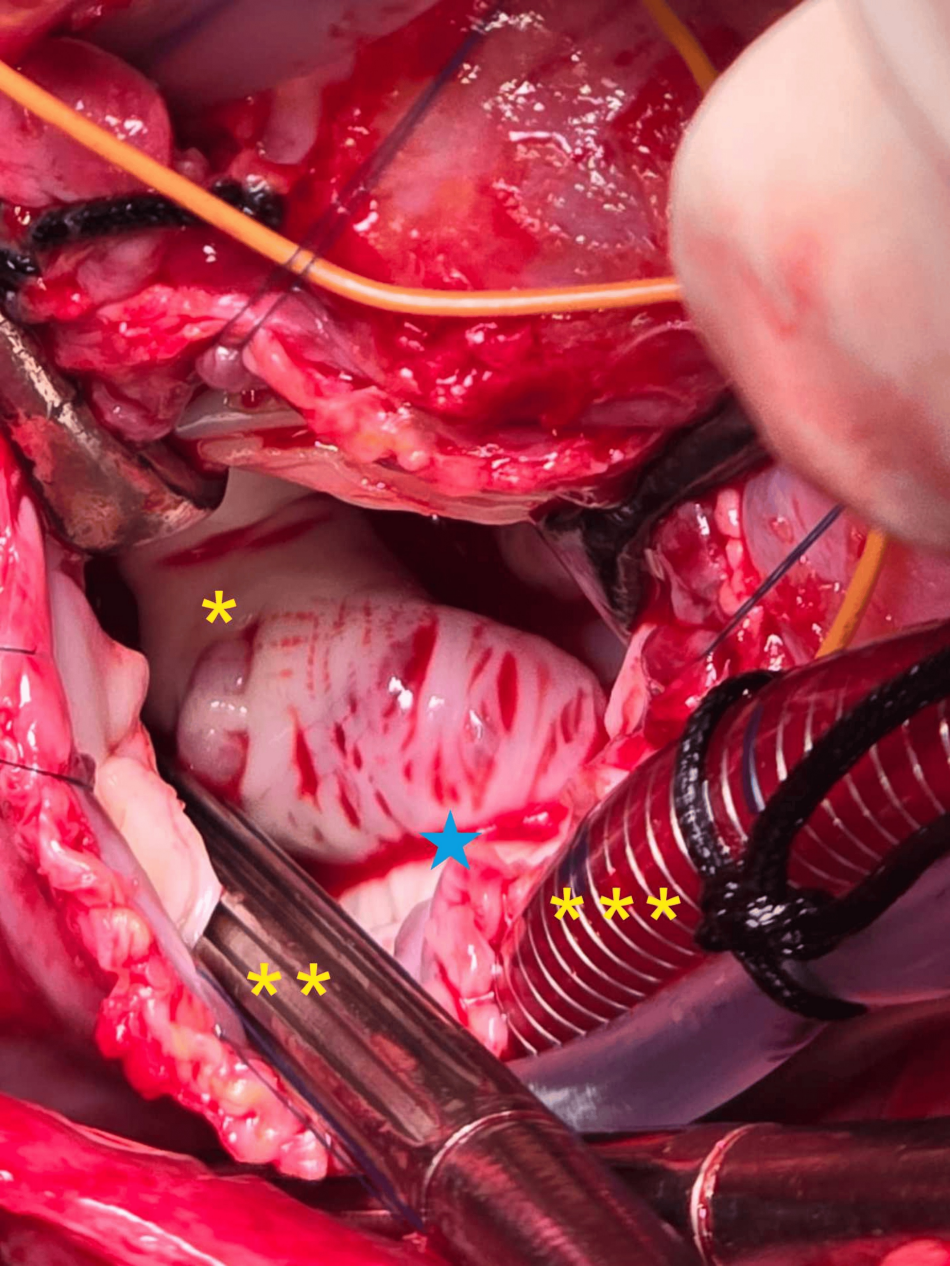
Intra-operative photograph from the surgeon’s perspective The left atrium is opened, with no thrombus observed in the left atrial cavity. The asterisk (*) denotes the left atrial cavity; the double asterisk (**) indicates the sump sucker within the left atrial cavity; the triple asterisk (***) marks the venous cannula in the inferior vena cava; the blue star denotes the mitral valve. Image Credits: Dr. Shrividya Shrishakumar Rao

**Figure 3:**
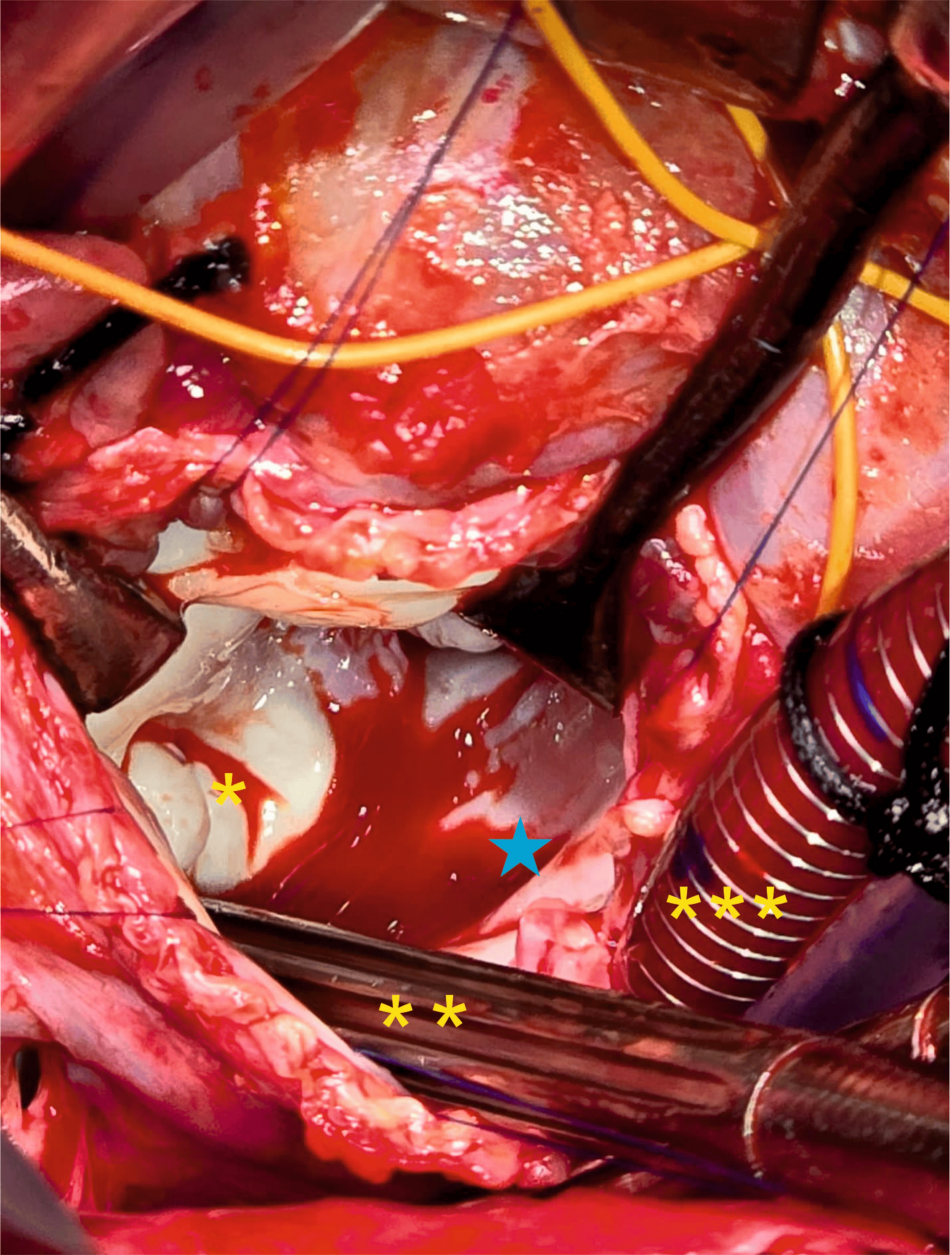
Intraoperative photograph from the surgeon’s perspective with the left atrium further retracted The inverted left atrial appendage is visible, projecting into the cavity. The asterisk (*) indicates the tip of the inverted appendage; the double asterisk (**) denotes the sump sucker within the left atrial cavity; the triple asterisk (***) marks the venous cannula in the inferior vena cava; The blue star denotes the mitral valve. Image Credits: Dr. Shrividya Shrishakumar Rao

A decision was made to proceed with reoperation to remove the mass and address the associated mitral valve pathology. Intraoperative TEE findings were consistent with those observed during the ICU TTE (Figures 1-3).

Standard hypothermic CPB (28 °C) was initiated with aorto-bicaval cannulation. Following cardioplegic arrest, the RA was opened, and the interatrial septum was incised to gain exposure to the LA and mitral valve. Intraoperative findings revealed an inverted, elongated LAA obstructing mitral valve inflow. No thrombus or vegetation was present. The mitral valve leaflets were inspected intraoperatively and were structurally and functionally intact. No direct leaflet pathology was identified. The LAA was everted, externally ligated, and clipped entirely outside the LA to achieve complete occlusion. External ligation was selected over internal exclusion or excision to avoid additional atrial incision and minimize operative time. No regurgitation was observed at the mitral valve following saline insufflation of the left ventricle. The patient was successfully weaned off CPB without complications. Postoperative TEE revealed no intracardiac mass or mitral valve stenosis or regurgitation. The patient was extubated on postoperative day 3, with complete resolution of pulmonary edema. She remained hemodynamically stable and asymptomatic during her subsequent hospital stay. Repeat TTE prior to discharge confirmed unobstructed mitral inflow and no recurrence of mass.

Consent to publish this case report was granted by the patient’s family following approval from the institutional ethics committee. Efforts were made to protect the patient’s identity.

## Discussion

Inversion of the LAA following pediatric and adult open-heart procedures is a rare complication, with 11 cases initially identified through a PubMed and Google Scholar search using the terms "inverted left atrial appendage", "LAA inversion", and "left atrial mass post-cardiac surgery", limited to English-language case reports [1-5]. A summary of these and two additional relevant reports encountered during manual reference cross-checking is presented in Table 1 [1 -13].

**Table 1:** Reported cases of inverted left atrial appendage following open-heart surgery ASD, atrial septal defect; CPB, cardiopulmonary bypass; ICU, intensive care unit; LA, left atrium; LAA, left atrial appendage; LVAD, left ventricular assist device; PDA, patent ductus arteriosus; PFO, patent foramen ovale; RA, right atrium; TEE, transesophageal echocardiography; TTE, transthoracic echocardiography; TOF, tetralogy of fallot; VSD, ventricular septal defect

Author, Year	Patient Age (Sex, if reported)	Diagnosis and Procedure	Recognition of Left Atrial Mass	Management	Outcome
Cohen et al., 1999 [1]	17 months	TOF, TOF repair	Post-bypass TEE	Reinstitution of CPB, cardioplegic arrest, LA opened, appendage everted.	Post-bypass TEE revealed no mass in the LA. The patient's postoperative course was uneventful, and he was discharged on post-operative day 10, at which time an echocardiogram showed no LA mass and good TOF repair with no RVOT gradient.
Troise et al., 2003 [2]	18 months	ASD and PDA, ASD closure with PDA ligation	Postoperative day 3, TTE	Conservative management with serial echocardiography and anticoagulation.	Discharged on oral anticoagulant treatment for a period of 6 weeks and antiplatelet therapy for 6 months. At 14 months of follow-up, he was found to be in good clinical condition with no symptoms of cardiac imbalance and/or clinical systemic embolism.
Aronson et al., 1992 [3]	56 years, female	Left ventricular thrombosis post-myocardial infarction, ventriculotomy, and thrombectomy	Post-bypass TEE	Reinstitution of CPB, cardioplegic arrest, LA opened, external eversion of appendage.	Successful outcome after two attempts at cardiopulmonary bypass (CPB)
Slavik et al., 1994 [4]	2 months, female	VSD, VSD closure with bovine pericardium	TTE in the ICU four hours after surgery	Reinstitution of CPB, cardioplegic arrest, LA opened, appendage ligated.	Her early postoperative course was uncomplicated, and she was discharged on day 10, with normal cardiac function on echocardiogram
Ekanem et al., 2020 [6]	57 years, male	Chagas disease with left ventricular failure, HeartMate III LVAD implantation	Postoperative day 3, TTE	Re-exploration, LVAD speed reduction, hydration, spontaneous eversion, AtriClip device occlusion of the appendage base	Unremarkable postoperative course with eventual discharge from the hospital
Buttar et al., 2022 [7]	3 years, female	ASD, ASD closure with bovine pericardium	Post-bypass TEE	Reinstitution of CPB, cardioplegic arrest, LA opened, appendage everted.	The post-operative course was uneventful, and the patient was discharged on the fourth post-operative day (POD). The follow-up TTE after the 4^th^ and 21^st^ day revealed no LA mass, and LAA was seen in its anatomical position.
Minich et al., 1995 [9]	5 months, female	Type 1 truncus arteriosus, VSD closure, and conduit repair	Postoperative day 2, TTE	Reinstitution of CPB, cardioplegic arrest, LA opened, external eversion, base ligated.	Successful outcome, unnecessary anticoagulation was avoided.
Arya et al., 2009 [10]	60 years, male	Coronary artery disease, coronary artery bypass grafting	Post-bypass TEE	Reinstitution of CPB, cardioplegic arrest, LA opened, appendage everted.	Not reported.
Choudhury et al., 2015 [11]	Adult, male	Aortic stenosis and dilated aortic root, Bentall procedure	Post-bypass TEE	CPB weaning and left atrial filling attempted, failed; external eversion with forceps.	Successful outcome after two attempts at CPB, the tip of the LAA was grasped with Russian tissue forceps and reverted to its normal anatomic position, which was confirmed with a repeat TEE examination.
Miyata et al., 2018 [12]	51 years, male	Mitral regurgitation, minimally invasive mitral valve repair via right thoracotomy	TEE during deairing	Spontaneous eversion following left atrial filling during CPB weaning.	The post-operative course has been uneventful, and the patient was discharged 7 days after surgery.
Liaqath et al., 2024 [13]	3 months, male	VSD, porcine pericardial patch closure of VSD	Post-bypass epicardial echocardiogram	Reinstitution of CPB, cardioplegic arrest, RA and septum opened, suction catheter eversion.	The baby recovered well following the surgery, was extubated within 24 hours, and had an uneventful recovery with no recurrence of appendiceal inversion. He is currently thriving well without any cardiovascular concerns.

This case is noteworthy in comparison to previous reports for several reasons: the patient was an infant post-VSD closure with progressive mitral inflow obstruction, delayed symptomatic deterioration, and successful management via external ligation and clipping, a technique less commonly used in the pediatric population. Additionally, preoperative TTE raised suspicion of inversion, and spontaneous eversion was not attempted due to the patient’s worsening hemodynamic instability and established mitral inflow obstruction.

Observing an echogenic structure in the LA is alarming in any postoperative setting, raising concerns about potential thrombotic events or infective endocarditis. Troise et al. reported a case of an inverted LAA identified on TTE after atrial septal defect closure on postoperative day 3 [2]. Similar findings were reported by Slavik et al. in an infant [4]. The patient was managed conservatively with follow-up and anticoagulation after excluding a prothrombotic state and infective endocarditis [2]. Cohen et al. highlighted that an inverted left atrial appendage (LAA), though rare, can mimic a pathological mass post-cardiac surgery, underscoring the importance of intraoperative recognition and timely correction to avoid misdiagnosis and unnecessary intervention [1]. Aronson S et al. reported similar findings in the intraoperative period [3].

Seven reports have described cases of inverted LAA appearing as a suspicious mass above the mitral valve (MV) on TEE performed in the operating room following surgery. These cases were managed by reinstituting CPB and manually everting the LAA [3,5,7]. Ankersmit et al. documented a female aged 38 years with Graves’ disease and partial seizures, with no history of cardiac interventions, who was diagnosed with a stroke [5]. Evaluation with TTE and magnetic resonance imaging of the heart revealed a mass in the LA suspected to be a myxoma. However, a de novo inverted LAA was identified and resected during surgery. Buttar SN et al. reported the occurrence of LAA after atrial septal defect correction [7].

The primary concern in these cases is the location of the LAA mass within the LA. When situated near the MV, the mass may trigger arrhythmias, thrombosis, infective endocarditis, necrosis, or even rupture during the postoperative period, which may necessitate urgent reoperation. Ekanem et al. reported a case of LAA as the cause of right heart failure in a patient with a left ventricular assist device (LVAD) [6]. In the present era of increasing usage of LVAD, it is imperative to consider this as the potential cause for deterioration in heart function. Arya et al. made this observation post coronary artery bypass surgery [10].

Minich et al. reported LAA presenting as a mass in the LA [9]. Miyata et al. observed LAA mimicking as a left atrial myxoma and findings during mitral valve repair [12]. Liaqath Ali et al. highlighted that inverted LAA can mimic thrombus in infants post-cardiac surgery [13].

Vella et al., in their computational modeling study, described multiple morphologies of the LAA and identified that inversion was most likely to occur with windsock and chicken-wing morphologies, especially under left atrial pressures lower than 5 mmHg [8]. Their model showed that inverted appendages could remain trapped if atrial filling was delayed or incomplete, providing a mechanistic explanation for cases of persistent inversion. Clinically, this highlights the importance of gradual de-airing and cautious use of suction during CPB. Several anatomical and procedural risk factors have emerged, including excessive left atrial suction, vigorous de-airing, and lack of LAA ligation during index procedures. The inverted LAA can mimic thrombus or vegetation, but its mobility, crescent shape, and lack of attachment to typical thrombus-prone zones (e.g., atrial septum, free wall) may help differentiate it. In our case, inversion was suspected based on the mass’s characteristic movement and location adjacent to the anterior mitral leaflet, although definitive diagnosis required surgical exploration [9-11].

Choudhury et al. emphasized that inverted LAA is a rare but preventable complication associated with excessive negative pressure during de-airing maneuvers in cardiac surgery, and recognition through intraoperative imaging can prevent misinterpretation as an intracardiac mass [11].

Approaches to managing an inverted LAA vary. Some clinicians take a conservative approach after ruling out more serious complications, monitoring patients with anticoagulation therapy [2]. When detected intraoperatively via TEE, waiting to see if complete filling of the heart prompts spontaneous eversion has been attempted. In other cases, reinstituting CPB for external manual eversion or inducing cardioplegic arrest for internal eversion has been necessary. In our case, spontaneous eversion was not attempted due to clinical deterioration, acute pulmonary edema, and imaging consistent with fixed inflow obstruction, warranting urgent surgical intervention.

Due to the rarity of this complication, no consensus exists on preventing LAA inversion or determining the best management approach if it occurs. External ligation, external clipping, and internal occlusion following cardioplegic arrest have all been successfully employed. However, standardized guidelines for preventing recurrence or determining optimal management have yet to be established.

## Conclusions

We presented a case of an inverted LAA observed after undergoing surgery to repair a VSD in an infant, which led to mitral valve inflow obstruction and acute pulmonary edema. The patient’s condition improved following surgical intervention involving external ligation and clipping of the LAA, resulting in both anatomical and symptomatic resolution. This case highlights the importance of considering LAA inversion as a differential diagnosis when a suspicious mass is detected in the LA postoperatively. Early recognition and prompt management can prevent serious complications, underscoring the need for heightened awareness and careful intraoperative and postoperative imaging evaluation. This case also suggests that systematic intraoperative inspection of the LAA, especially in pediatric cardiac surgeries, should be considered as part of imaging checklists to reduce misdiagnosis and guide appropriate surgical decision-making.
